# Matrikines in kidney ageing and age-related disease

**DOI:** 10.1097/MNH.0000000000000916

**Published:** 2023-08-16

**Authors:** Alexander Eckersley, Tomohiko Yamamura, Rachel Lennon

**Affiliations:** a Division of Musculoskeletal & Dermatological Sciences, School of Biological Science; b Wellcome Centre for Cell-Matrix Research, School of Biological Science, Faculty of Biology, Medicine and Health, The University of Manchester; c Department of Paediatric Nephrology, Royal Manchester Children's Hospital, Manchester University Hospitals NHS Foundation Trust, Manchester, UK

**Keywords:** basement membrane, collagen IV, extracellular matrix, matrikines

## Abstract

**Purpose of review:**

Matrikines are cell-signalling extracellular matrix fragments and they have attracted recent attention from basic and translational scientists, due to their diverse roles in age-related disease and their potential as therapeutic agents. In kidney, the matrix undergoes remodelling by proteolytic fragmentation, so matrikines are likely to play a substantial, yet understudied, role in ageing and pathogenesis of age-related diseases.

**Recent findings:**

This review presents an up-to-date description of known matrikines with either a confirmed or highly anticipated role in kidney ageing and disease, including their point of origin, mechanism of cleavage, a summary of known biological actions and the current knowledge which links them to kidney health. We also highlight areas of interest, such as the prospect of matrikine cross-tissue communication, and gaps in knowledge, such as the unexplored signalling potential of many kidney disease-specific matrix fragments.

**Summary:**

We anticipate that knowledge of specific matrikines, and their roles in controlling processes of kidney pathology, could be leveraged for the development of exciting new future therapies through inhibition or even with their supplementation.

## INTRODUCTION

The extracellular matrix is a highly diverse network of macromolecular proteins including collagens, glycoproteins, and proteoglycans. These molecules concomitantly act as an organized scaffold between cells and within connective tissue. In the kidney, matrix assemblies not only support nephron structures within the glomerulus and tubulointerstitium by conferring physical rigidity and elasticity [1] (e.g. collagen I/III and fibrillin-1), but also play a crucial role within the glomerular basement membrane, where organized layers of collagen IV and laminin networks allow the filtration of small molecules from the blood into the urine [2].

Although matrix best known for its physical properties, the importance of matrix signalling, and its control of tissue and systemic homeostasis is becoming increasingly recognized and demanding further investigation. Matrix proteins are capable of controlling cell behaviour in a number of ways, such as through changes in local biomechanical stiffness [3], direct interaction between cell receptors and specific amino acid sequences [4] (e.g., integrins and the RGD (Arg-Gly-Asp) sequence) and even sequestration, storage and release of growth factors and cytokines [4] (e.g., elastic fibre-storage of TGF-beta by latent TGF-beta binding protein [5] and heparan sulphate proteoglycan-binding of Wnts and hedgehog [6]). However, it is the proteolytic release of cell-signalling fragments from the matrix that has attracted recent attention from basic, translational and even cosmeceutical scientists, due to the diverse roles of these fragments in age-related disease and their potential as therapeutic agents. These bioactive matrix fragments have been termed matrikines [7].

In the kidney, matrix undergoes remodelling either through progressive degeneration in ageing, such as in the glomerular basement membrane, or aberrant accumulation, as seen in chronic kidney disease (CKD) associated with fibrosis [8]. The mechanisms behind these changes can be partly attributed to the dysregulation of proteases and their inhibitors, such as the induction of matrix metalloproteases (MMPs), and the recruitment and activation of fibroblasts, all of which leads to a loss of equilibrium between matrix degradation and deposition [9]. Due to the propensity for matrix fragmentation during these processes, it is likely that matrikines play a substantial, yet understudied, role in ageing and pathogenesis of age-related kidney diseases.

This review presents an up-to-date description of known matrikines with either a confirmed or anticipated role in kidney ageing and disease, including information on their protein of origin, their mechanism of cleavage and a summary of the current knowledge behind their biological action. Although many proteolytic matrix fragments exist which may serve as potential biomarkers of kidney ageing and disease, this review will predominantly focus on those with confirmed signalling capabilities (Table 1). These molecules could be considered true matrikines, and therefore most likely contribute to pathogenesis and functional tissue decline and would therefore most benefit from future study. 

**Box 1:**
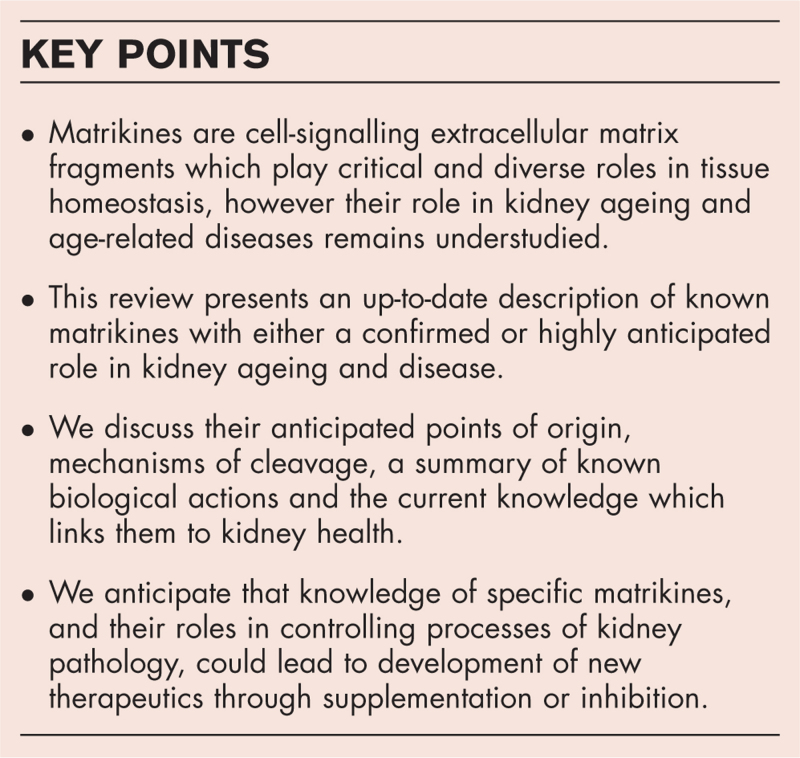
no caption available

**Table 1:** List of matrikines with either a confirmed or anticipated role in kidney ageing and age-related disease. Only ECM fragments with confirmed signalling capabilities *in vitro* or *in vivo* are shown.

Parent Protein	Matrikine	Originating protein region	Molecular Weight	Amino acid position	Cleaving enzyme	Functions	Expression in kidny	Role in kidney	References
Collagen I	p1158/1159	C terminal end (α1)	∼3.5 kDa	1159–1196	MMP2, MMP9	Promoting angiogenesis promoting fibrosis	Probably yes	not known	[11,12^▪▪^]
	PICP	Trimerised C terminal propeptide (α1, α2)	120 kDa	α1: 1208–1453 α2: 1126–1372	MMP2, MMP9	Promoting migration in endothelial cell	Probably yes	not known	[13]
Collagen IV	Arresten	NC1 domain (α1)	14 kDa	1445–1669	MMP3, MMP9, MMP15	Antiangiogenic antitumour promoting apoptosis in endothelial cell	Yes	elevated in AKI mouse kidney	[14–24]
	Canstatin	NC1 domain (α2)	26 kDa	1486–1712	MMP3, MMP9, MMP15	Antiangiogenic antitumour promoting apoptosis in endothelial cell	Yes	elevated in AKI mouse kidney	[14,15,19–24]
	Tumstatin	NC1 domain (α3)	24 kDa	1426–1670	MMP9	Antiangiogenic antitumour promoting apoptosis in endothelial cell	Yes	decreased in renal cancer cell	[25–27]
	Tetrastatin	NC1 domain (α4)	28 kDa	1465–1690	Unknown	Antiangiogenic	Probably yes	not known	[28–30]
	Pentastatin	NC1 domain (α5)	∼2.5 kDa	1516–1650	Unknown	Antiangiogenic	Probably yes	not known	[28,30]
	Hexastatin	NC1 domain (α6)	∼2.5 kDa		Unknown	Antiangiogenic antitumour	Probably yes	not known	[28,30]
	PN4P 7S	7S domain (α1, α2)	14 kDa	28–172	Unknown	Promoting migration in neutrophil	Probably yes	elavated in blood of patients with HD potential marker of kidney fibrosis	[1,34,33]
Collagen VI	Endotorophin	C5 domain (α3)	8–99 kDa	3112–3162	BMP-1	Promoting angiogenesis promoting fibrosis promoting tumour growth	Yes	elavated in urine/blood of patients with CKD potential marker of CKD	[35–37,38^▪▪^,^39^]
Collagen XV	Restin	NC1 domain (α1)	18∼22 kDa	1198–1386	BMP-1 (not confirmed)	Antiangiogenic antitumour	Yes	not known	[45,46]
Collagen XVIII	Endostatin	NC1 domain (α1)	20 kDa	∗COL18A1 has 3 isoforms, and all isoforms secrete Endostatin	MMPs, cathepsins, elastase	Antiangiogenic antifibrotic antitumour	Yes	inhibits diabetic kidney disease in mouse model increased expression in aged mouse kidney Overexpression induces kidney fibrosis	[40–44]
Multiple collagen	PGP (proline-glycine-proline)	ubiquitous	269 Da		MMP8, MMP9, prolyl endopeptidase	Chemoattractant for neutrophils	Not confirmed	not known	[47,48]

BMP, bone morphogenetic protein 1; CKD, chronic kidney disease; MMP, matrix metalloproteases.

## MATRIKINES AND THEIR FUNCTIONS

There are 28 different collagens in the human genome and the most intensely investigated are fibrillar collagens (e.g., type I and type III) and the basement membrane type IV collagen [10]. These collagens have associated matrikines with a range of biological actions. In addition to collagens, various other matrix proteins, such as glycoproteins (e.g., fibulin-1) and matricellular proteins, are now known to produce matrikines and have attracted recent attention.

## COLLAGENS

### Type I collagen

Type I collagen is one of the most abundant structural matrix proteins, forming a heterotrimer with two α1 chains and one α2 chain, and it is widely distributed throughout the body, including the kidneys. Peptides produced by the degradation of type 1 collagen by MMPs have been reported to be involved in several physiological processes, such as angiogenesis [11] and matrix remodelling [12^▪▪^]. Polypeptide fragments found at (and referred to by) their starting amino acid position at p1158/159, produced through cleavage by MMP2 and MMP9 from the C-terminal region of mature type I collagen α1 chain and further degraded by MMP9 into smaller fragments, are thought to act as matrikines [11]. Studies where the first 15 amino acids of p1158/159 were purified have reported that it promotes wound healing both in fibroblasts and in a mouse model of myocardial infarction by accelerating matrix remodelling and angiogenesis [11]. This small peptide also improved vascular re-endothelialization, collagen fibre deposition and organization by inducing remodelling of the matrix in a mouse model of vascular injury [12^▪▪^]. Furthermore, a trimer of C-terminal-derived peptides (propeptide trimer carboxyl-terminal to type I collagen: PICP), which are cleaved and released from the precursor type I procollagen during the formation of type I collagen α1 and α2 chains, have been shown to induce directional migration of vascular endothelial cells in vitro [13].

### Type IV collagen

Among the matrikines that have attracted much attention in recent years, the peptide fragments derived from type IV collagen are the oldest known and studied for their independent physiological effects. Type IV collagen is a major component of basement membrane along with laminin networks in all tissues and forms triple helix structure in combination of six subunits (α1–α6 chains). The chains assemble into the triple helix trimers composed of alpha chains (α1α1α2, α3α4α5 and α5α5α6) and then to hexamers where the NC1 domains of the trimers interact. The hexamers then form polymer networks by interactions between 7S domains. While the α1α1α2 network is widely distributed in basement membrane of all tissues, α3α4α5 is localized predominantly to kidney, lung, eye and inner ear basement membranes. The α5α5α6 network is present in the skin, smooth muscle and is enriched in Bowman's capsule in the kidney. Type IV collagen contributes not only to maintain the structure of basement membrane, but also to cell adhesion, migration, proliferation and differentiation via interaction with various cell membrane receptors such as integrins. In recent years, the degradation products of the noncollagenous domain of collagen IV by matrix degrading enzymes such as MMPs has attracted attention as novel bioactive peptides. Peptide fragments derived from the NC1 domain of the α1 to α6 subunits are called Arresten (α1), Canstatin (α2), Tumstatin (α3), Tetrastatin (α4), Pentastatin (α5) and Hexastatin (α6), respectively.

Arresten is a 26 kDa polypeptide derived from the α1 chain and produced by the action of MMP3, MMP9 [14] and MMP15 [15]. Arresten has been reported to exhibit antiangiogenic and antitumour effects by inhibiting vascular endothelial cell proliferation, migration and promoting apoptosis via integrin α1β1 both in vivo and in vivo [16–18]. Canstatin is a 24 kDa peptide produced from the α2 chain through cleavage by MMP3, MMP9 [14] and MMP15 [15] and, like arresten, exhibits antiangiogenic activity by inhibiting vascular endothelial cell proliferation, migration and lumen formation. It is also known to exhibit antitumour effects by inducing apoptosis of tumour cells [19,20]. A previous study has suggested that these effects are mediated by suppression of the downstream signalling pathways focal adhesion kinase (FAK)/phosphoinositide-3-kinase (PI3K)/Akt through interaction with α_v_β_3_ and α_v_β_5_ integrins [21]. It has also been reported that the administration of recombinant canstatin into a rat model of pulmonary hypertension suppressed right ventricular remodelling such as cardiomyocyte hypertrophy and interstitial fibrosis in the right ventricle, suggesting a protective effect on cardiac cells [22]. Arresten and canstatin are known to be up-regulated in the kidneys of mouse models of ischaemia-induced acute kidney injury, suggesting that they may be involved in the pathogenesis of kidney disease [23,24].

Tumstatin is a 28 kDa matrikine cleaved from the α3 chain of type IV collagen by MMP-9 and has been reported to inhibit the proliferation and induce apoptosis of vascular endothelial cells and tumour cells [25]. The angiogenesis inhibitory activity of tumstatin is localized to the Tum-5 domain, consisting of a 54–132 amino acid region which mediates angiogenic activity by binding to the αVβ3 integrin in an integrin binding site (RGD) independent manner [25,26]. Tumstatin has been reported to be down-regulated in kidney cancer tissue [27] though, tumstatin expression is also known to be altered in other tumorigenic diseases. Therefore, the specific pathological significance of this matrikine in the kidney is still unknown.

Compared to these type IV collagen α1–α3 chain-derived matrikines, there are fewer studies on the α4–α6 chain-derived tetrastatin, pentastatin and hexastatin. The NC1 domain of the α6 chain (hexastatin) has long been known to have angiogenesis inhibitory and tumour suppressive properties. However, the products of the α4 and α5 chains were not found to have similar effects in the same study [28]. A subsequent study found that the tetrastatin (with a size of 28 kDa) from the α4 chain could inhibit melanoma cell proliferation and invasion [29]. Furthermore, a novel peptide of 19–20 residues derived from the NC1 domain of α4–α6 exhibited antiangiogenic effects by inhibiting the proliferation and migration of vascular endothelial cells in vitro [30]. The role of these peptides in the kidney, in both healthy and diseased states, has not been specifically reported. However, further studies are crucial, as the α3α4α5 and α5α5α6 chains are major components of the basement membrane of glomeruli and Bowman's capsule [31^▪▪^,^32^].

Besides peptides derived from the NC1 domain, the N-terminal 7S domain of type IV collagen is also known to act as a matrikine with physiological activity. It has been reported that the type IV collagen 7S domain promotes the migration of neutrophils [33]. The amino-terminal propeptide of its procollagen form (P4NP) is cleaved off during conversion from type IV procollagen to type IV collagen and is suggested to increases in plasma reflecting systemic collagen IV formation. In patients with end-stage kidney failure on haemodialysis, plasma PN4P levels have been reported to correlate significantly with mortality [34] and are expected to be a new marker for CKD with kidney fibrosis [1].

### Type VI collagen

Type VI collagen is a beaded filament collagen and is expressed as a ubiquitous matrix protein in the interstitium, forming a microfibrillar network associated with the basement membrane. Endotrophin, which is released from the C-terminal C5 domain of the α3 chain by bone morphogenetic protein 1 (BMP-1) [35], has attracted much attention as a promising matrikine. Adipocyte-derived endotrophin increases fibrosis, angiogenesis and inflammation, and promotes tumour growth by mobilizing macrophages and endothelial cells through enhanced TGF-β signalling in breast cancer tissue [36]. It has also been revealed to act as a stimulator of the TGFβ pathway in adipose tissue during consumption of high-fat diet intake, causing fibrosis and inflammation and ultimately increasing insulin resistance [37]. In the kidneys, type VI collagen is increased in CKD with fibrosis, alongside alterations of kidney matrix in ageing and disease [31^▪▪^]. Accumulated collagen VI is thought to release endotrophin and promote fibrosis. According to an observational study, endotrophin is increased in the urine and blood of patients with IgA nephropathy and ANCA-associated vasculitis, and has been shown to correlate with the degree of fibrosis in kidney tissue [38^▪▪^]. Based on these results, it is expected to be a marker to predict the progression of CKD [39].

### Other collagens

In addition to those described above, several other collagen-derived matrikines have been studied. Restin and endostatin are C-terminal fragments of the noncollagenous domains of types XV and XVIII collagens respectively which exhibit high sequence homology. Endostatin, the C-terminal fragment of type XVIII collagen, is cleaved by MMPs, cathepsin and elastase and has a size of 20 kDa [40]. It inhibits endothelial cell growth via cyclin D1 inhibition and induces apoptosis. Through this mechanism, endostatin has been shown to inhibit tumour growth in various tumour cells [41]. Although endostatin is mainly expected to have antitumour therapeutic applications, it has also been reported to inhibit the progression of diabetic kidney disease in a mouse model of diabetes [42]. Furthermore, renal expression of endostatin was shown to be elevated in aged mice [43] and its overexpression induces interstitial fibrosis [44], indicating a potential role in kidney ageing and age-related disease. Restin also inhibits endothelial cell migration and inhibits angiogenesis [45] and has antitumour effects [46]. PGP, a tripeptide cleaved from various collagens by MMP8 and MMP9, is known to act as a neutrophil attractant via binding to CXC chemokine receptors [47,48].

## OTHER KIDNEY MATRIX PROTEINS

### Fibulin-1

Fibulin-1 is a matrix glycoprotein and widely expressed in various tissues, including the cardiovascular system, lung, skin and kidney. In recent years, fibulin and its degradation products have attracted attention for their physiological activities and potential as markers for various diseases. In particular, fibulin-1C (FBLN1C1), the degradation product of fibulin-1, is known to promote the adhesion and proliferation of lung fibroblasts [49].

### Testican-2

Encoded by *SPOCK2,* testican-2 is a matrix protein and is expressed by podocytes [50]. It binds glycosaminoglycans and localizes to basement membranes [51]. Testican-2 has glycosaminoglycan attachment sites at the C-terminus and contains thyroglobulin type-1, follistatin-like, and calcium-binding domains. It was identified as a podocyte-derived marker of kidney health [50] and functional studies demonstrated that testican-2 increased glomerular endothelial tube formation and cell motility in vitro. A subsequent study showed that testican-2 levels were associated with kidney health in three cohorts including more than 8000 individuals [52^▪▪^]. However, the specific region of this 47 kDa protein that confers the risk or protection is unclear and whether testican is a true matrikine is yet to be determined.

## FUTURE PERSPECTIVES AND CONCLUSION

**FIGURE 1:**
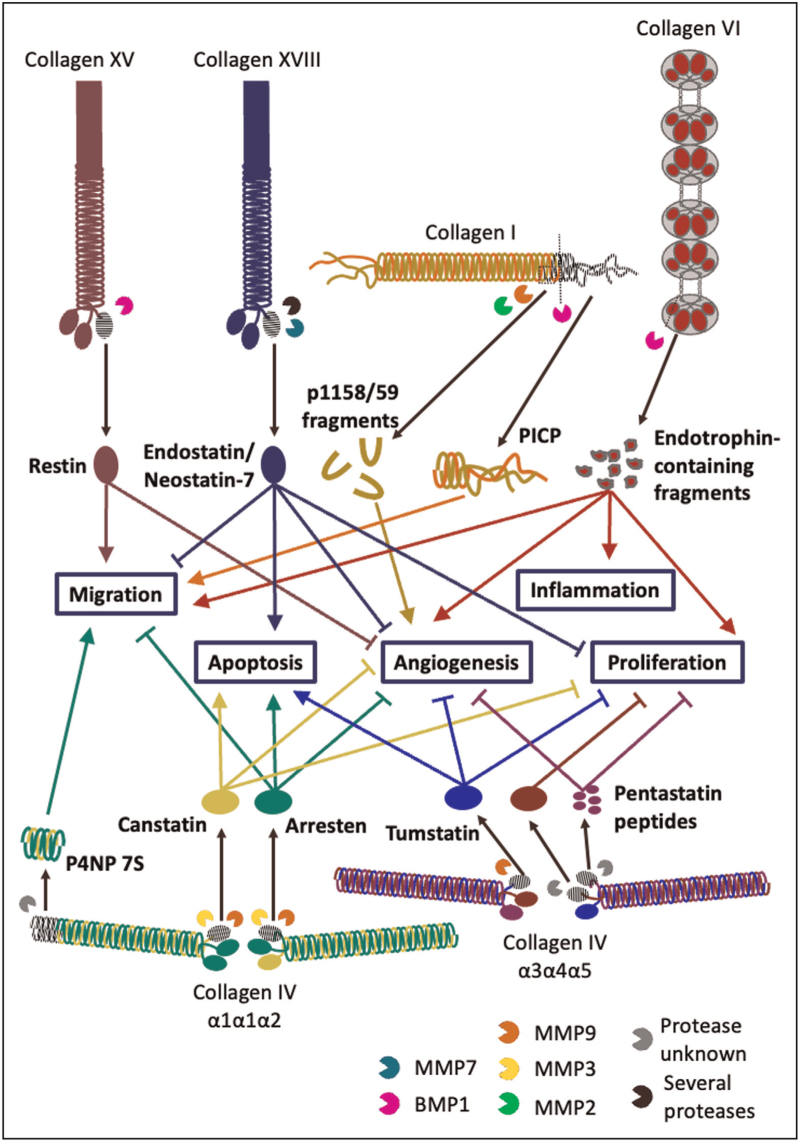
Several tissue collagens undergo proteolytic fragmentation by endogenous proteases (e.g., MMPs and BMP1), sometimes during normal synthesis (e.g., the release of PICP and endotrophin from type 1 procollagen and type VI collagen α3 respectively) but often during matrix remodelling. Some of the fragments released are known to bind to distant cells and elicit changes in behaviour. These matrix fragments with cell signalling capabilities are termed matrikines, which can modulate a myriad of cell responses, from proliferation, migration and angiogenesis (processes crucial for tumorigenesis) to apoptosis and inflammation. It is highly likely that aberrant matrix remodelling observed in kidney ageing and CKD leads to the release of several different matrikines that contribute to its runaway disruption of homeostasis and functional decline. BMP, bone morphogenetic protein 1; CKD, chronic kidney disease; MMPs, matrix metalloproteases.

We have highlighted several matrix fragments from collagens and other matrix proteins with confirmed roles as bioactive matrikines, and which may therefore contribute to kidney health, ageing and disease (collagen matrikines are summarized in Fig. 1). It is important to note that a plethora of kidney matrix components, including basement membrane laminins [53] and the proteoglycans agrin [54] and versican [55], are vulnerable to proteolytic fragmentation *in vivo* and that these fragments may yet play undetermined roles as matrikines in health and disease. Further research into the biological action of these fragments and how they orchestrate kidney homeostasis and disease mechanisms is required, opening up the field to promising new avenues of study.

Another promising new field of research is the potential role of matrikines in cross-tissue communication. Several of these bioactive fragments can enter the bloodstream (and often serve as serum biomarkers of renal disease [34,38^▪▪^]), potentially at vascular endothelial basement membranes such as within the glomerulus and alveolus [56^▪▪^], leading to speculation that matrikines may exist as part of a feedback loop capable of mediating multiorgan function with implications in ageing and disease. This concept has been recently explored by Jandl *et al.*[57^▪▪^] in association to pulmonary-renal syndromes (such as Goodpasture's). They propose that basement membrane and associated matrix fragments, generated for example through injury or degeneration during alveolar haemorrhaging and glomerulonephropathy, may be released in circulation where they then act as danger associated molecular pattern (DAMP) signalling molecules which bind and elicit extra-organ changes in cell response and immune cell activation.

A challenge that still exists in the field of matrikine research is the ability to characterize matrix fragmentation events and release of matrikines *in vivo*. Novel proteomic methods, such as peptide location fingerprinting which is capable of measuring changes across protein structures, have the potential to identify the fragmentation and release of matrikines which could facilitate future research [56^▪▪^,^58^].

As our understanding about the biological role of matrikines grows, so too will their use as biomarkers of tissue health, regulation of tissue homeostasis and disease and their roles in cross-tissue communication become clearer. We anticipate that matrikine regulation (through inhibition or supplementation) will enable exciting new therapies in future.

## Acknowledgements

*None.*

### Financial support and sponsorship

*This work was supported by a Wellcome Senior Fellowship awarded (202860/Z/16/Z) to R.L. and supporting A.E. and a JSPS Overseas Research Fellowship supporting T.Y.*

### Conflicts of interest

*There are no conflicts of interest.*

## REFERENCES AND RECOMMENDED READING

Papers of particular interest, published within the annual period of review, have been highlighted as:

▪ of special interest
▪▪ of outstanding interest
